# Development of an Elliptical Perturbation System that provides unexpected perturbations during elliptical walking (the EPES system)

**DOI:** 10.1186/s12984-023-01251-3

**Published:** 2023-09-26

**Authors:** Shoval Sade, Hodaya Pickholz, Itshak Melzer, Amir Shapiro

**Affiliations:** 1 Department of Mechanical Engineering, Faculty of Engineering, Ben-Gurion University of the Negev, Beer-Sheva, Israel; 2 Schwartz Movement Analysis & Rehabilitation Laboratory, Physical Therapy Department, Faculty of Health Sciences, Recanati School for Community Health Professions, Ben-Gurion University of the Negev, P.O.B. 653, Beer-Sheva, Israel

**Keywords:** Balance reactive responses, Falls, Perturbation-based balance training, Old people

## Abstract

**Background:**

‘Perturbation-based balance training’ (PBBT) is a training method that was developed to improve balance reactive responses to unexpected balance loss. This training method is more effective in reducing fall rates than traditional balance training methods. Many PBBTs are performed during standing or treadmill walking which targeted specifically step reactive responses, we however, aimed to develop and build a mechatronic system that can provide unexpected perturbation during elliptical walking the **E**lliptical **Pe**rturbation **S**ystem (the EPES system), with the aim of improving specifically the trunk and upper limbs balance reactive control.

**Methods:**

This paper describes the development, and building of the EPES system, using a stationary Elliptical Exercise device, which allows training of trunk and upper limbs balance reactive responses in older adults.

**Results:**

The EPES system provides 3-dimensional small, controlled, and unpredictable sudden perturbations during stationary elliptical walking. We developed software that can identify a trainee’s trunk and arms reactive balance responses using a stereo camera. After identifying an effective trunk and arms reactive balance response, the software controls the EPES system motors to return the system to its horizontal baseline position after the perturbation. The system thus provides closed-loop feedback for a person’s counterbalancing trunk and arm responses, helping to implement implicit motor learning for the trainee. The pilot results show that the EPES software can successfully identify balance reactive responses among participants who are exposed to a sudden unexpected perturbation during elliptical walking on the EPES system.

**Conclusions:**

EPES trigger reactive balance responses involving counter-rotation action of body segments and simultaneously evoke arms, and trunk reactive response, thus reactive training effects should be expected.

## Introduction

As lifespan increases, much more older adults are at risk of falling [1] Deficits in sensorimotor system among older adults affect muscle strength and power [2, 3], balance control [4–6] walking abilities [7, 8], and reactive balance [9, 10], which may increase the risk of falls and require physical intervention. Falls may have severe consequences that can cause injuries such as broken bones or a head injury [11, 12]. About 3 million older people are treated every year in emergency departments in the US, and about 1000 older adults in Israel visit emergency departments due to a fall every day. Over 800,000 older adults in the US are hospitalized as a result of a head injury or hip fracture for fall injuries [1]. The data shows that more than 95% of hip fractures are caused by falling sideways [13, 14]. Furthermore, falls have a serious financial burden [15] and psychological impact [16, 17].

Balance intervention programs were found to be effective to reduce fall rates in older adults. A recent Systematic review [18] found in high-certainty evidence that compared with control group, balance and functional exercises reduce the rate of falls by 24% (RaR 0.76, 95% CI 0.70 to 0.81) among 7920 older adults, in 39 RCT studies. In regard to other types of exercise, there is moderate-certainty evidence that balance, functional exercises that also included resistance exercises, reduce the rate of falls by 34% (RaR 0.66, 95% CI 0.50 to 0.88) among 1374 older adults who participated in 11 RCT’s. Low-certainty evidence was found in regard to Tai Chi exercises that was found to reduce the rate of falls by 19% (RaR 0.81, 95% CI 0.67 to 0.99) in 2655 older adults, in 7 RCT’s. The above training programs involve preplanned anticipatory postural adjustment functional exercises. But most falls happen during unexpected balance loss [19]. Therefore, Perturbation-Based Balance training (PBBT) was developed. The PBBT is a specific type of balance training where participants frequently exposed to unannounced balance losses, aimed to evoke and improve balance reactive responses to avoid a fall [20, 21]. A recent meta-analysis [22] found in a moderate-certainty evidence that older adults who assigned to reactive balance training groups reported 40% fewer falls than control groups (rate ratio: 0.60, 95% confidence interval = [0.42, 0.86]; p = 0.005, I2 = 83%). Thus, it seems that PBBT has a better result than conventional balance training program in reducing falls, since it simulates unexpected balance loss that closely mimics real-life situations. The prevalence of adverse events of PBBT was somewhat higher compared to control groups (29% vs. 19%; p = 0.018), 79 of 271 PBBT participants reported adverse events (mild: n = 63, moderate: n = 15, severe: n = 1) compared to 51 reported adverse events in 263 control participants (mild: n = 43, moderate: n = 8).

Many PBBT’s are conducted by using a mechatronic system that provide external perturbations during treadmill walking [22–26]. These devices are designed to specifically train the change of support (i.e., reactive stepping responses) in older people who are able to stand or walk independently without external support, i.e., holding handrails [22–26]. These PBBT’s usually last 20–45 min twice or three times a week, 3-months to train lower limbs, trunk, and upper limbs balance reactive responses [22–24]. Many older adults are less able to participate in this type of PBBT. These include older adults that suffer from a severe lower limb osteoarthritis, having difficulty to be exposed to the high ground reaction impact forces that are induced during this type of training (e.g., walking and performing rapid stepping responses), indicates that such treadmill walking training is beyond the capacities of many older adults. This may present accessibility challenges for older adults with osteoarthritis of lower-limb joint to perform and benefit from PPBT methods. In addition, data suggest that controlling center of mass (CoM) motion after unexpected perturbations through trunk movement control is critical for balance maintenance and fall-prevention in older adults [27] and stroke survivors [28]. The ability to limit trunk motion has consistently distinguished laboratory-induced falls from successful recovery [29–31], lower trunk flexion angle and trunk velocity were associated with successful recovery after underfoot perturbations [31, 32, 32–36] and following a trip during overground walking [30]. Several studies reported that trunk reactions are highly involved in controlling reactive fixed-BoS strategies [37, 38] as well as in stepping reactions following different methods of forward, backward, and lateral perturbations [32–39]. Regarding the stepping responses, the trunk angle at foot contact is a strong predictor of successful recovery by a single-step reaction to a perturbation in AP and lateral directions [29, 32, 36, 40]. Moreover, the trunk angle at foot contact was also reported to be one of the principal mechanisms by which balance recovery is adapted with repeated exposure to balance perturbations [41, 42]. It was found that older adults participating in Perturbation Based Balance Training programs led to a significant reduction in the maximum trunk angle during balance recovery [34, 43]. Reducing trunk rotations will have a significant effect in bringing the CoM of the body within stability limits provided by the feet. Therefore, training-related improvements in reactive balance control after specifically providing feedback for trunk reactive responses. Given that the trunk parameters consistently reflect an effective reactive stepping, we developed the **E**lliptical **Pe**rturbation **S**ystem (the EPES system), that include perturbation training during elliptical walking to improve capabilities of trunk reactive responses. A randomized controlled trial will be conducted to investigate the effects of the proposed EPES training program on gait and balance function in older adults. This addresses two key issues in relation to interventions: (a) the importance of learned adaptations in response to external perturbations (i.e., concerning the task specificity) and (b) the shaping of perturbation training to the older adult’s neuromotor capacities in order to optimize training responses and applicability to real-world challenges (i.e., individualization). We believe that the proposed training technique will help participants to improve control trunk movements immediately after perturbations and effectively decelerate CoM motion over the base of support, thus improve balance reactive control and reduce number of falls.

Recent data suggests that Elliptical training offers advantages over other training tools, since its constant double-limb support diminishes recurring high-impact lower-limbs joint reaction forces while allowing exercise in a functional, upright posture. The results show lower joint pressure and force values training when exercising on elliptical compared with overground walking (40% and 43% lower, respectively) [44]. We assume that recovery step responses after perturbated walking would demonstrate even higher values. These findings suggest that the constant double-limb support during the elliptical training likely minimizes joint peak pressures. Therefore, development of elliptical device that perturbed subject during elliptical walking may provide PBBT that do not require exposure to high ground reaction forces impact to the lower-limb joints. In order to match the perturbation training approach for these people, aiming to specifically train the reactive trunk, hip, and arm balance response, designing and developing a mechatronic system that provides balance training that includes perturbations while elliptical walking can be valuable. We were also inspired by the fact that in-place walking, elliptical walking, and regular overground walking are lower-extremity rhythmic tasks with similar reflex modulation, and related neural circuitry may be operating in these tasks [45, 46]. In order to match the perturbation training approach for older adults with lower limb osteoarthritis, aiming to specifically train the reactive arms, trunk, as well as legs balance response, designing and developing a mechatronic system that provides balance training that includes perturbations while elliptical walking can be valuable.

We were also inspired by the well-known health advantages gained after adults participated in elliptical training, such as improvement in cardiovascular parameters [47], increasing muscle power and endurance [48], and improving executive function [49] and quality of life [50]. Moreover, elliptical training improves gait parameters in older adults with lower limb Osteoarthritis [51], people with stroke [52], Parkinson’s [53], multiple sclerosis [54], and people with cardio-respiratory disease [55, 56]. This is not surprising since elliptical walking is a rhythmic task with similar reflex modulation to a regular walking [45, 57, 58], and related neural circuitry may be operating in both tasks [46, 59].

In this paper, we aimed to describe the design, development, building, and clinical applications of a novel mechatronic perturbation-based **E**lliptical **S**ystem that provides unexpected perturbations during in-place walking (the EPES system). The system delivers 3-dimensional unannounced external perturbations **EPES** during elliptical walking (see details in the system description below). The **EPES** specifically challenges balance reactive responses during elliptical walking which is suitable for all older people but specifically for older adults with lower limb osteoarthritis. Osteoarthritis (OA) is the most common joint disorder in the United States [60], the risk of developing knee OA to be about 40% in men and 47% in women, And the prevalence is close to that [61, 62]. The number of people suffering from OA will only increase due to the aging of the population and the obesity epidemic [63]. In this paper we will also describe the development of a software that identify the trainee’s balance reactive responses which aimed of controlling the motor of the EPES system as well as provide feedback to the trainee and trainer in regard to a successful balance reactive response. Finally, we report the results of a pilot study that aimed to explore whether the software identifies balance reactive function during EPES elliptical walking.

## Methods

### System description

**Fig. 1:**
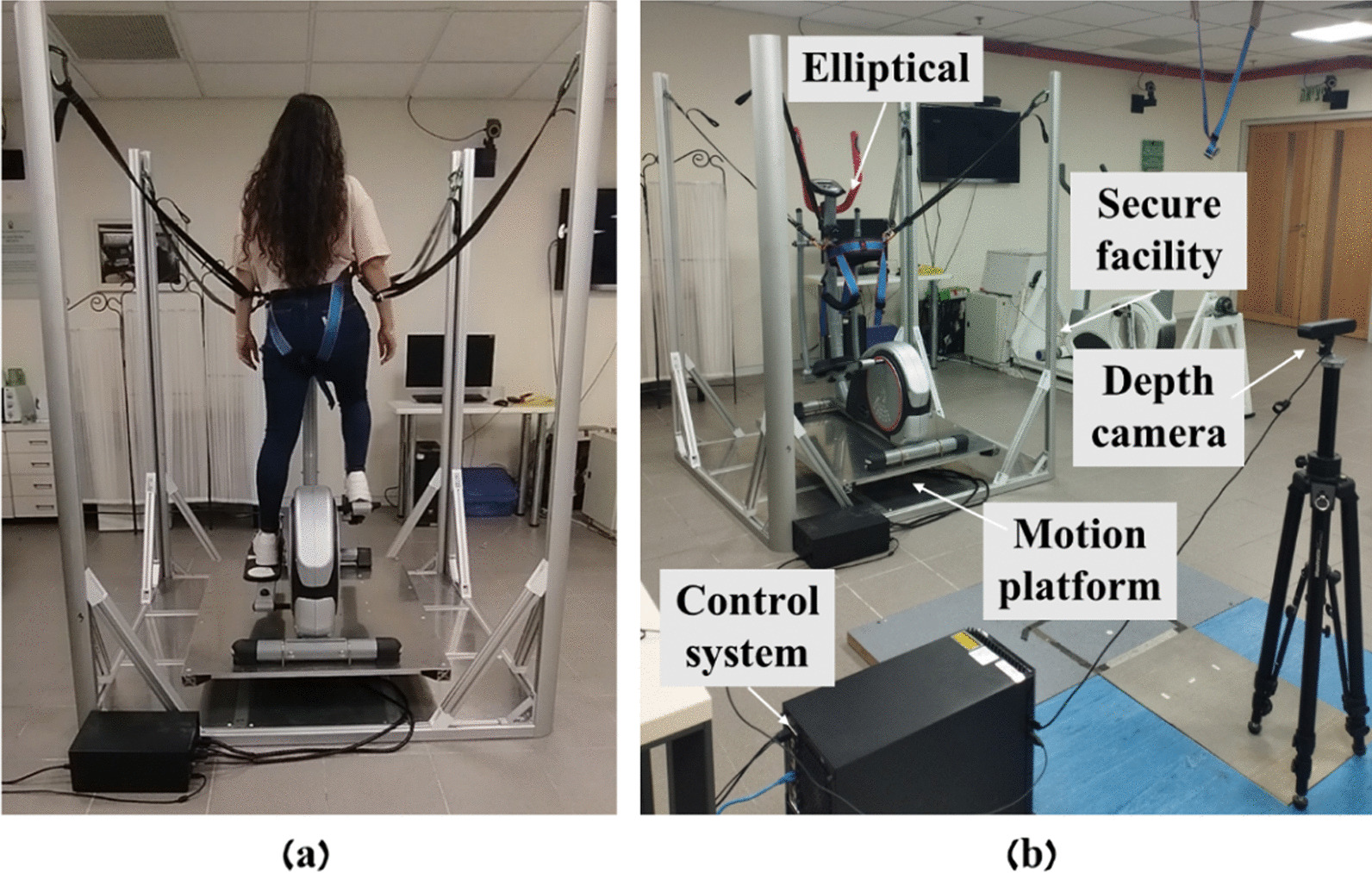
The final system. **a** Illustration of how the subject is connected to the safety harness system, **b** presentation of the system components

The EPES system is a mechatronic device weighing 140 kg that provides 3-dimensional (3D) balance perturbations tilts during in-place walking (see Fig. 1). According to the cost per item, the total price of the system is ~ 10,500$. Assembly, manpower and testing costs are not included in the price. In addition, the system is not yet in serial production, which affects its overall cost. It is comprised of a stationary elliptical system that is mounted on a motion platform that consists of two DC motors and gears connected to them. The motion platform allows two degrees of freedom, roll and pitch (left–right and forward–backward tilts, respectively) during elliptical walking in a safe environment. The EPES system provides a maximum 3D perturbation tilt angle of a maximum 8° (in all directions) with 3 options of rotational strength. The motor that performs the unannounced perturbation tilts is controlled by a motion control system which is controlled also by a camera system (ZED 2 from STEREOLABS), which are both controlled by a main computer software program. The computer program is on the host PC which also serves as a user interface. By a program command, the motion control system directs the motion platformer rotation based on the training plan. The computer software program allows the trainer to determine EPES perturbation parameters such as the tilt angle of perturbation, rotational strength, direction of rotation, and the interval time between perturbations.

Based on the ZED2 camera, the software can capture and identify effective balance reactive reactions after unannounced balance perturbation is given. In case an appropriate balance reactive response is detected by the software i.e., counter-rotation action of body segments, the tilt rotation (i.e., the perturbation) is stopped, and the motor returns the EPES system to its horizontal position (i.e., zero position) by motor counter-rotation. In this way, the trainee gets real-time feedback. The immediate real-time balance reactive response feedback may help the trainee to learn implicitly how to react successfully to unexpected perturbation and provides the best possible motor learning implementation [64].

### Main EPES system components

#### The motion platform

**Fig. 2:**
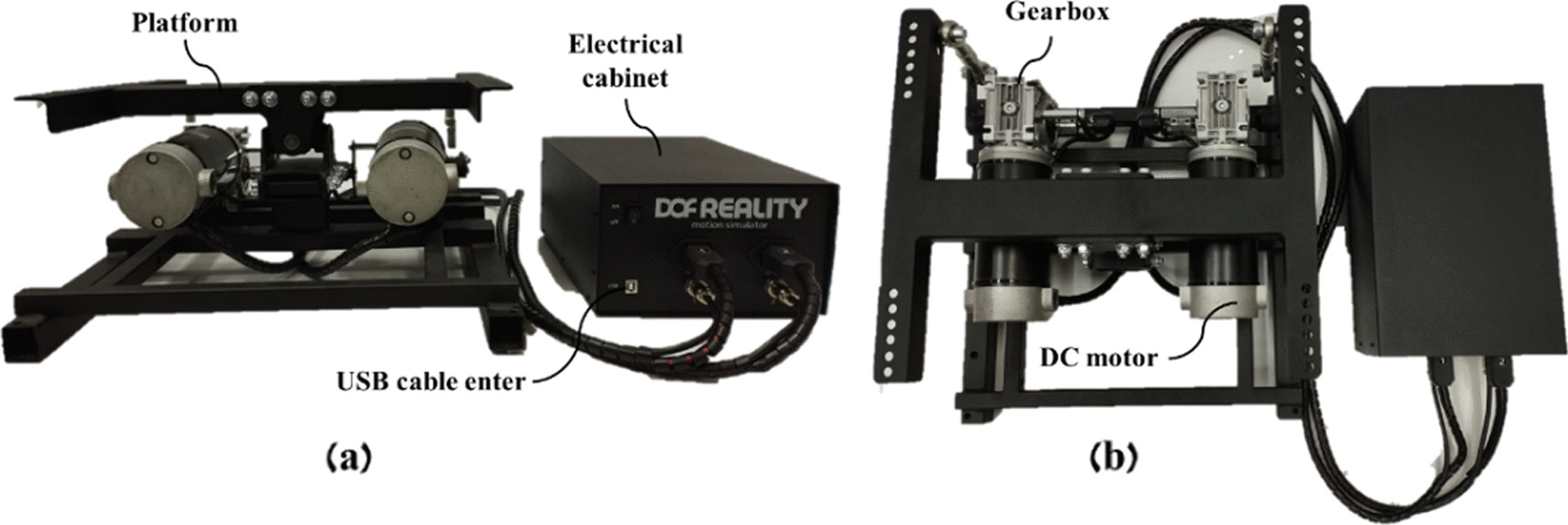
The motion platform system. **a** Front view, **b** top view

The motion platform is an off-the-shelf product purchased through the DOF reality company (dofreality.com). It is consisting of a platform (on which the elliptical is placed), two 24 V DC brushed motors and gears connected to them (see Fig. 2). To link the rotation of the motors to the tilt of the platform, a four-bar linkage mechanism is attached to each output shaft of the gear. This mechanism allows control over the position of a certain point in a closed kinematic system [65]. Thus, through rotation combinations of the motors, it is possible to control the tilting direction of the platform—left, right, forward or backward tilt. The two DC motors with a power rating of 200 W, and the gear is NMRV gear type with a gear ratio of 1:50. Also, the motors have a maximum speed of 105°/s and peak torque of 25 Nm. On the other side of the output shaft of the gear, there is a position sensor that monitors the rotation of each motor. The motion platform is controlled by an Arduino which is located inside an electrical cabinet (the motion platform controller) that came with the platform. The electrical cabinet connects with a USB cable to the PC for the purpose of connecting to an application programming interface (API) called Sim Raising Studio (SRS). This API enables programming of the motion platform using Python software in a convenient and simple way. Therefore, it is possible to determine the tilt angle and its direction. We are unable to determine the acceleration directly, but by using a python function we wrote, we can control the intensity of the tilt (slow, medium, fast—range between 1 and 3). As a rough estimate, the angular velocity at intensity 1 is 3°/s, at intensity 2°/s–8°/s, and at intensity 3°/s–16°/s. As well, the plate always returns to a horizontal position at a 2°/s.

#### The stereo camera

**Fig. 3:**
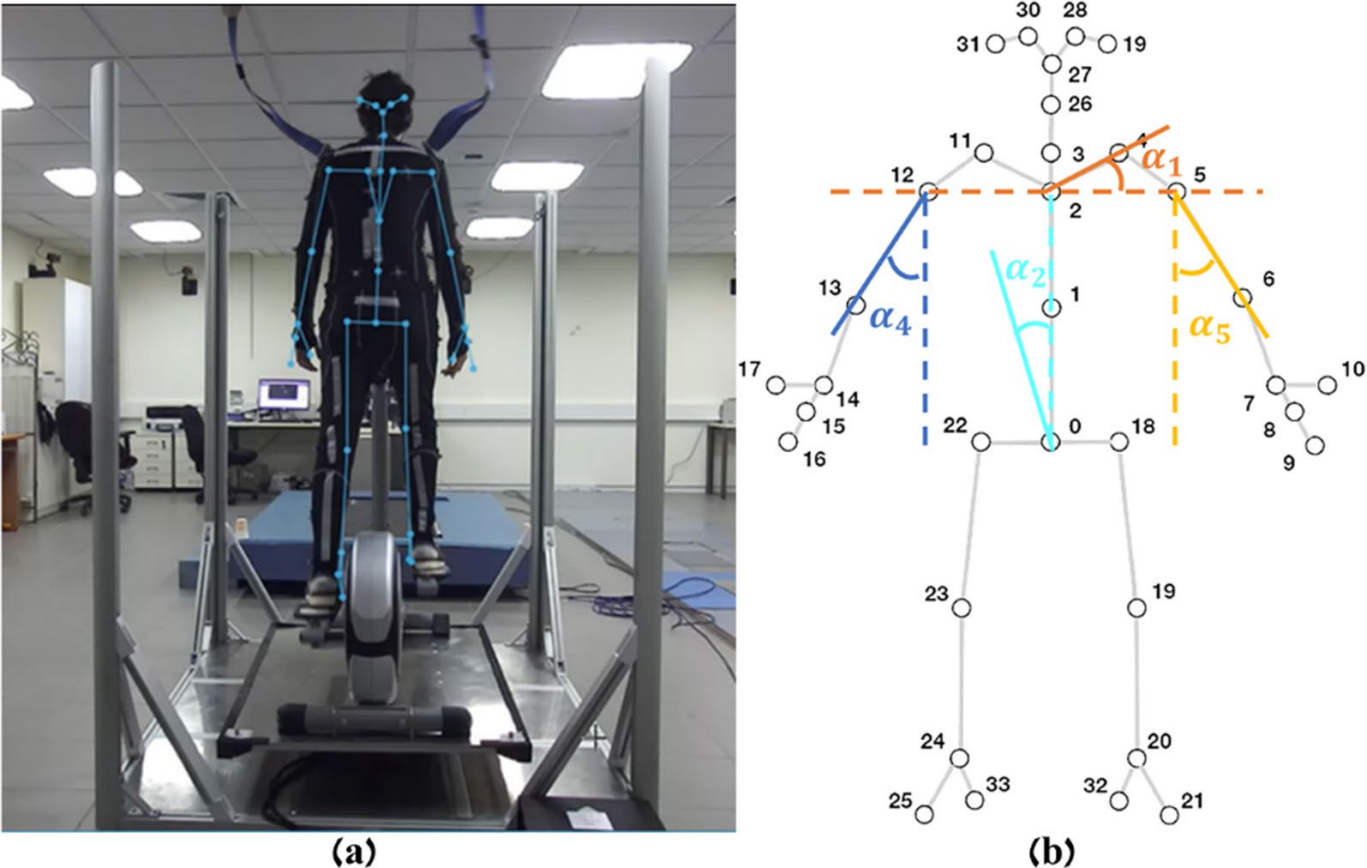
**a** The EPES software’s ability to monitor and identify the process of the participant’s sensorimotor adaptation to hands-fee pedaling (when the trainee connecting to the stationery secure system), **b** illustration of the defined angles (see text for further descriptions of joint angels)

The ZED 2 is a stereo camera that is mounted at a horizontal plane at a height of 1.05 [m] and 1.8 [m] in behind the trainee’s standing position for the best motion capture of the trunk and arms’ reactions. The ZED 2 camera is a depth camera in which there is a ready-made and easy-to-use body frame model that maps the human body into a single kinematic chain (see Fig. 3). This way the ZED 2 camera captures the body posture in real time and allows implementation of the upper-body balance reactive responses to be monitored and increased. The camera sensors collect the trainee’s body movements with respect to the EPES system state and analyze their responses to ongoing events.

**Fig. 4:**
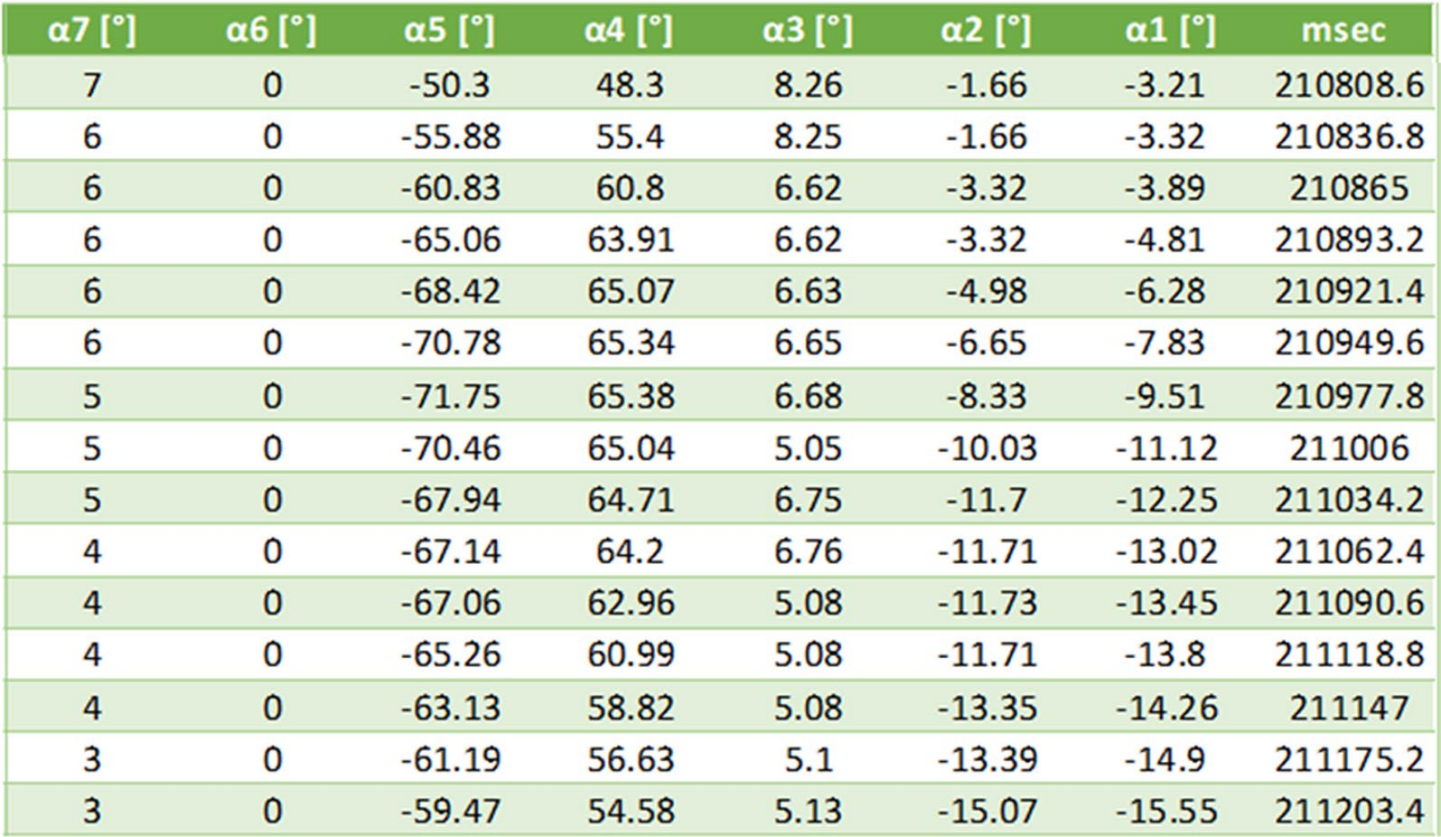
A section from the CSV file was opened using Excel software that includes the time and the different angles (α1–α7 in degree)

During training, the system calculates predefined angles (α1–α5 [°]) of the trainee’s body (see Fig. 3). The angles are calculated using the information received from the camera about the position of the trainee’s joints. We used the upper-body joints skeleton stick figure that the camera provides because the balance, trunk, and arm movements are the training target. While training, the system calculates the desired angles (α1–α5 [°]) and saves them in a CSV file (see Fig. 4) to be able to observe the trainee’s reactions and analyze them after training is over. Also, the system collects and saves the tilt angles (α6, α7 [°]) that are sent to the motion platform in a CSV file as well, when: α1) Shoulder angle: the angle of the line between the trainee’s two shoulders and the ground on the left–right axis; α2) L-R trunk angle: the angle of the line from the trainee’s center of mass (CoM) to the trainee’s chest relative to the line vertical to the ground on the left–right axis; α3) F-B trunk angle: the angle of the line from the CoM to the chest relative to the line vertical to the ground on the back-front axis; α4) Left hand angle: the angle between the line from the left elbow to the left shoulder joints of the trainee and the line vertical to the ground on the left–right axis; α5) right hand angle: the angle between the line from the right elbow to the right shoulder joints of the trainee and the line vertical to the ground on the left–right axis; α6) system roll angle: the angle of the motion platform on the left–right axis; α7) system pitch angle: the angle of the motion platform on the front-back axis. The angle of the motion platform was defined relative to the horizontal plan, a positive angle indicates forward or rightward downward tilts, and a negative angle indicates backward or leftward downward tilts. Similarly, a positive trunk or shoulder angle indicates a forward or rightward body tilt, and a negative angle indicates a backward or leftward body tilt. As explained above, the hands’ angles are calculated in relation to an axis perpendicular to the ground. A positive angle indicates clockwise rotation, while a negative angle indicates anticlockwise rotation. Thus, most of the time, right hand angles will be positive and left-hand angles will be negative.

Each training consists of two phases: calibration and perturbation. First, by calibrating, we identify the effective balance response threshold during in-place walking on the elliptical personalized for each trainee. Secondly, during the perturbation phase, when the trainee responds well to perturbations, the system provides a customized intrinsic sensorimotor cue. This is done by stopping the perturbation immediately and returning the EPES system to its horizontal position. We used angles α1 and α2 in the real-time training process to detect the trainee’s body position with respect to the calibration angles to check if an effective balance reactive response was performed when the system performs roll (left–right) perturbation. We used angle α3 for the same purpose when the system performs pitch (forward–backward) perturbation. Angles α4 and α5 were not used during the real-time training process but are shown in post-training graphs for advanced post-training analysis. Monitoring the trainee’s balance responses according to the system movement over time can indicate the implementation of skill acquisition and the motor learning progress of the balance upper-body reactive responses. The data of angles α1–α5 reveals to the trainer all the information about the balance reactions. For example, the arm reactions (α4 and α5), which are part of the training goal, are reflected in all types of perturbations. But the other angles that are measured represent more reliable information about the nature of the response according to the perturbation; Therefore, the calculation is not based on these angles. However, it can clarify the entire response to perturbations.

#### The control system (PC)

The computer program serves as the control system and runs on the host PC. Using Python software, it is possible to control both systems, the ZED 2 camera, and the motion platform, and even to communicate between them. Python software activates both systems simultaneously. Thus, the system knows the position of the user and, as a result, the angles of his body due to the ZED 2 camera. And the desired perturbation can be created while controlling the motion platform. The motion platform controlled thanks to its API (SRS) as mentioned earlier. With the help of Python software, you can send simple commands to the SRS for the type of perturbation, the size of its angle and its intensity. The SRS receives the information and sends the translated information to the controller of the motion platform that takes care of care of moving the motors according to the command sent.

#### Safety harness

Safety is an extremely important issue since, in this training, we apply unexpected perturbations that may cause older people to fall off the EPES system. A safety harness keeps the trainee secure during training. For the safety system there are two options to secure the trainer according to the nature of the required system: mobile or stationary.

##### For mobile system (see Fig. 1a)

A waist harness is used to secure this system. Four adjustable straps are attached to this harness, two on each side (right and left), when each of which is fixed to one of the four side rods of the system. This system is mobile because it does not depend on the site on which it is placed.

##### For stationary system (see Fig. 3)

A body harness is used to secure this system. This safety harness is suspended from the ceiling by two ropes above the trainee. This system is stationary because it is necessary to fix the straps to which the harness is attached to the ceiling of the site where the system is placed. The experimenters were secured to this system.

In both systems, the harness is slightly loose to be safe, and not restrict balance response, but in case the trainee fails to recover, the safety harness will arrest the fall.

#### Software

Currently, the program can be activated with Python software and the trainer can select the type of training by answering questions at the beginning of the training run.

##### Creating training

When running the program, at first the system asks the trainer to enter the length of the workout in minutes. This time includes the first 2 min of system calibration. Following that, the trainer defines training content by defining segments of perturbations based on his preferences. Each perturbation is defined separately and added in chronological order to the training. For each segment of perturbations, the trainer sets the duration of the segment, the type of the perturbations (the tilting direction, the size of its angle, its intensity (range between 1 and 3), and the delay time between each two consecutive perturbations (frequency). Defining segments ends when all the defined training minutes have been used. After creating the training, the system is activated, and the trainee is instructed to start walking on the elliptical. In the first 2 min of the training, the EPES is calibrated according to the trainee trunk motion, so there are no perturbations in this part. The purpose of this part is to define the range of user trunk and shoulders angles (mean and standard deviation) when the user walks on the system while it is at rest (no perturbations). After this time, the perturbations begin according to the training trunk and shoulders segments that the trainer created. Also, the system saves the calculated trunk and shoulders angles of the trainer and the system angles perturbations in a CSV file. Note: the system saves in a CSV file the system and user angles during the training phases.

### Types of perturbations

The EPES system provides 3D tilting balance perturbations that aim to challenge specifically trunk, and upper body balance reactive reactions but also lower-limb reactive responses are triggered. When tilting, the trainee’s CoM aside rapidly, the trainee is forced to decelerate the CoM by responding reactively with lower-limbs, trunk and upper limb balance reactive response during elliptical walking. Balance perturbations are provided in two forms: (1) machine-induced unannounced external perturbations and (2) self-induced internal perturbations during hands-free elliptical walking. The external perturbations are controlled programmed machine-induced and are ranged from low to high magnitude (0°–8° for each direction). They can be programmed expectedly as a block perturbation training (fixed time, order, and magnitude), or be given unexpectedly as random perturbation training (in onset, direction, and time interval) which evoke fast trunk and upper-body reactive balance motion. The internal self-induced perturbations are provided by the trainee during self-pace elliptical walking on the EPES. These situations fall under proactive balance control training when the trainee shift his/her body weight during the elliptical walking.

### EPES system communication and activation

**Fig. 5:**
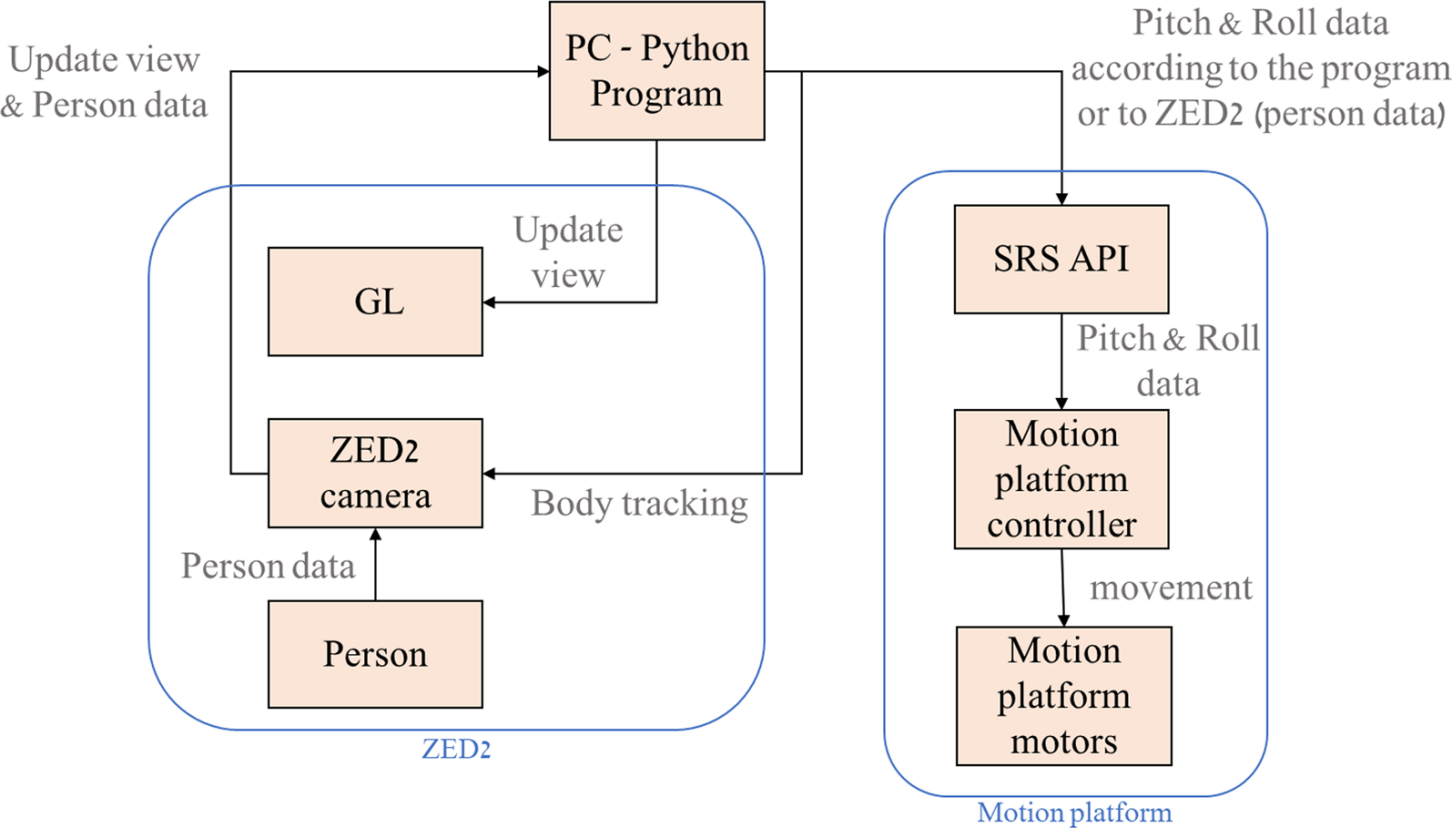
The EPES communication flow chart

To activate the EPES system first you need to make sure that the PC has access to the motion platform controller, i.e., turn on the motion platform controller and open the SRS application. Next, for running a training program, the trainer first creates a customized perturbation training program with the user interface (activate Python software). When the computer program runs the training program by utilizing the motion control system and the ZED 2 camera, both controlled by the computer program. It is also necessary to enable communication between the two systems (the camera and the motion platform) to produce implicit feedback for the trainee. The camera transmits information about the trainee’s shoulder trunk and arms movements, while the motion control system is responsible for receiving information and delivering commands to the motors. In case the software detects an appropriate trunk balance reactive response during a perturbation i.e., counter rotating the trunk and shoulder, the perturbation is stopped, and returns to its horizontal position. When the software doesn't detect an appropriate trunk balance reactive response, the system will continue to tilt until it reaches the defined angle and remains there until it detects an effective balance reactive response. Also, with the help of the graphics library (GL), the system displays the camera image with the body frame (body sticks diagram) throughout the training session. The desired mode of activation and communication between the camera and the motion platform is shown in Fig. 5.

After the training program, the trainer may review the trainee’s balance reactive movement graphs, check if an effective reactive response was triggered and balance was recovered, and the data collected in order to determine how to proceed in the next training session.

### The 3D camera function during the training

**Fig. 6:**
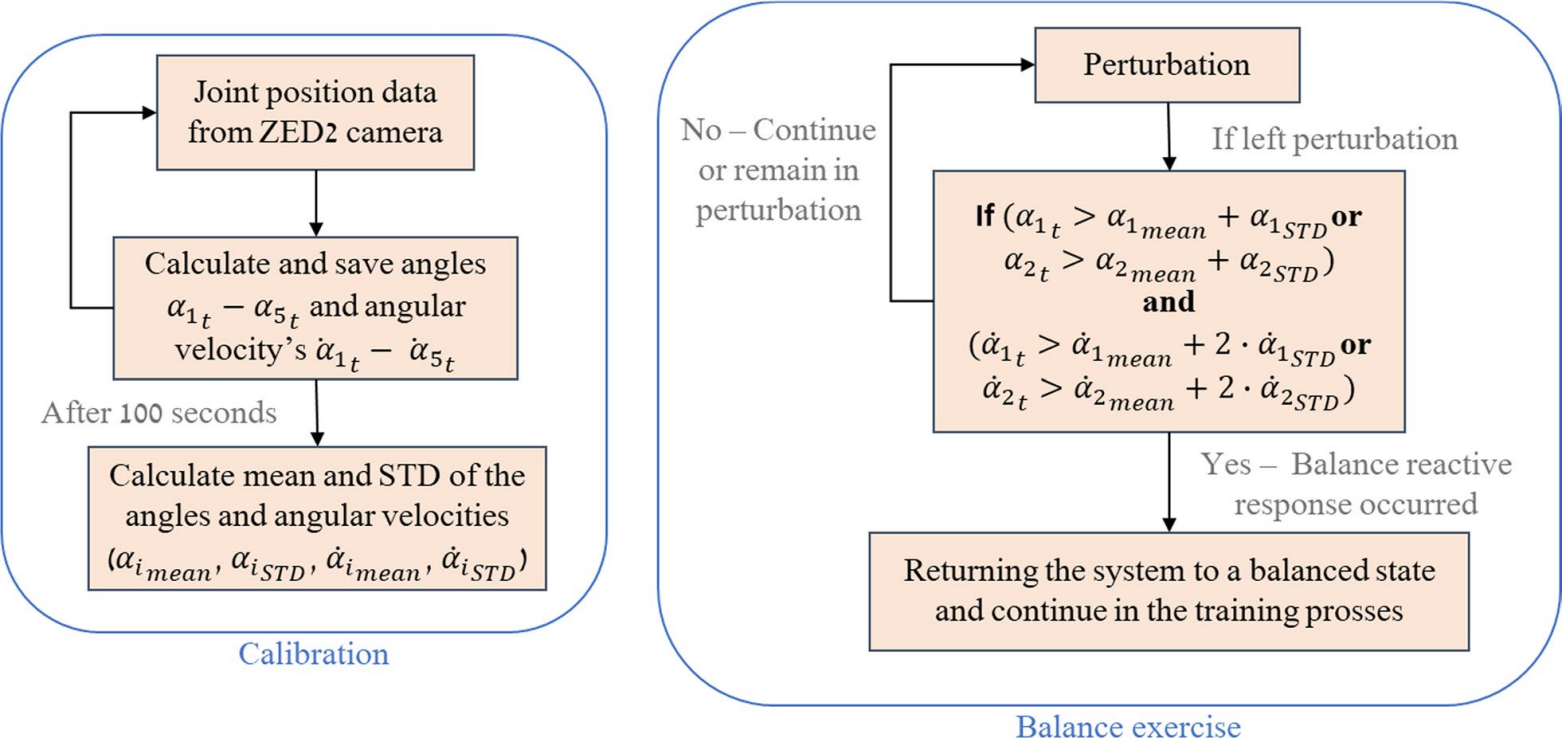
Calibration time and checking the user’s response during left perturbation flow charts

The training session is divided into two stages: (1) the calibration stage (the first 2 min) and (2) the balance exercise stage (see Fig. 6).

1. **The calibration stage**—for automatically customizing the EPES system to the trainee who is currently using it. It consists of two parts:
  A) The trainee’s adaptation phase—20 s of warning-up slow pedaling to allow the trainee to ease into a comfortable position. In this phase, the computer program does not make any reference point calculations due to the noisy data that was gathered by the camera until the trainee gets used to walking on the elliptical.
  B) Measuring and calculating each individual upper body sway (forward–backward and left–right) trunk angle; the body sway base noise range (natural angles)—100 s of self-paced pedaling while the ZED 2 camera provides data to the computer program for calculating angles α1-α5. In this phase, first, the computer program records the data of the angles α1–α5 during the 100-s of self-paced elliptical walking. Secondly, based on all the angles calculated, the software calculates each angle and angular velocity’s mean and standard deviation (STD)—the “normal” angles and angular velocities range. This information will be used later to determine whether the trainee’s reactive balance responses were effective or not will be obtained. Based on the “normal” angles and angular velocities range, the computer program detects if the trainee responded to a given perturbation or whether their current trunk and shoulder angle was a part of natural movement during pedaling (i.e., into the body-sway-base-noise range). The natural movements are the angles at which the trainee is naturally walking in place, and this is necessary because often older people naturally tend to lean a few degrees to either the left, right, forward, or backward side.
2. **The balance exercise stage**—contains block and expected or random and unexpected balance perturbations. When a new perturbation is executed, the computer program compares between the trainee’s trunk and shoulder angles and angular velocities (which are calculated and kept continuously throughout the training) to the angles and angular velocities range during the self-induced internal perturbations during hands-free elliptical walking. This is to see if there has been a significant movement other than a pedaling movement.

For separating a balance reactive response to a normal self-induced voluntary pedaling movement following a perturbation the system programed to check these conditions: If the EPES motion platform angle and the trainee’s body are leaning in opposite directions, and if the current body angle and angular velocity is larger than the natural angle and angular velocity of this side self-induced pedaling. This tilt requires the trainee to have a larger distinct balance reactive response to recover their balance for passing above the response threshold and stopping the perturbation by turning the EPES angle back to its neutral position (horizonal to the ground). This option deals with a trainee who exhibits large trunk and shoulders angles during the exercise session versus the calibration phase. Figure 6 shows algorithms for the calibration phase and the balance exercise phase with an emphasis on perturbation appearance time. In the exercise phase, an example of a situation where the perturbation is to the left (negative EPES angle) is presented. In this case, the software check is to determine if the subject is tilted to the right (positive angle).

### Exploring balance reactive responses

Here, we present results of a proof-of-concept pilot study to explore whether the EPES software was able to identify balance reactive responses. Two people young adults were exposed to unannounced right–left and anterior–posterior balance perturbations during elliptical walking on the EPES system. The tilting perturbations evoked balance reactive trunk, head, and arm movements almost always in the opposite direction of the perturbation to quickly move the upper body’s CoM toward the base of support provided by the EPES pedals. Both participants performed upper body balance reactive responses during the training session.

**Fig. 7.**
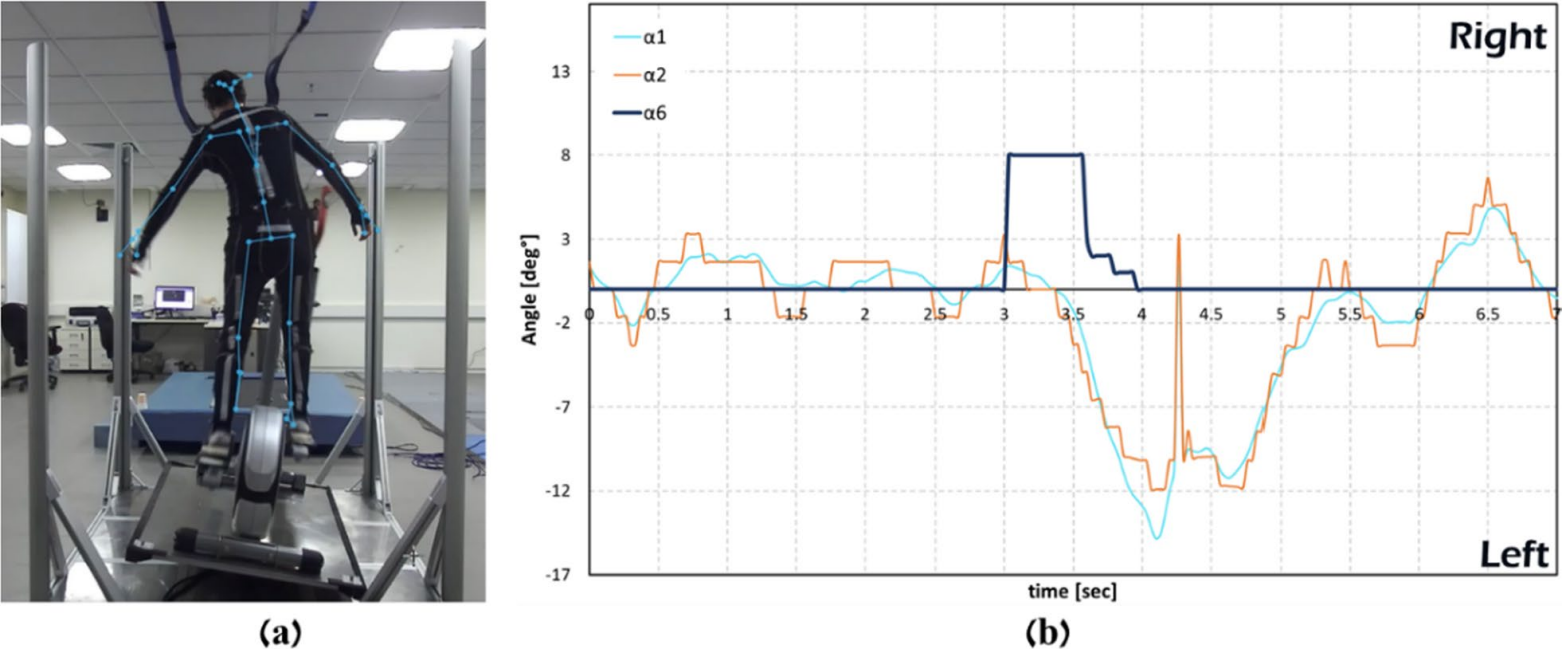
8° right tilt perturbation. **a** Camera image during the experiment, **b** a graph created from the saved CSV file. The system roll angle increases by 8° (dark line, α6), followed by a large balance recovery reaction as seen by the 12° trunk angle (orange line, α2) and about 14° shoulder angle (light blue line, α1) is, as clearly seen in **B**. Immediately after an effective balance recovery response was detected by the software, the EPES system returns to its initial horizontal condition (dark line, α6)

The trunk, shoulder and arms reactive responses during the training sessions is demonstrated in Figs. 7 and 9. Upper bodies balance reactive responses are presented by the shoulder line and trunk/back. These angles were found to be the best parameters to distinguish the presence of an upper-bodies balance reactive response. In Fig. 7, the participant pedaled without holding the elliptical system handlebars (i.e., hands-free), and exposed to 8º right announced perturbations during pedaling (Fig. 7A). Figure 7B shows that the EPES software was able to accurately identify shoulder and trunk balance reactive responses and return the system to its initial horizontal plane (Fig. 7B).

**Fig. 8.**
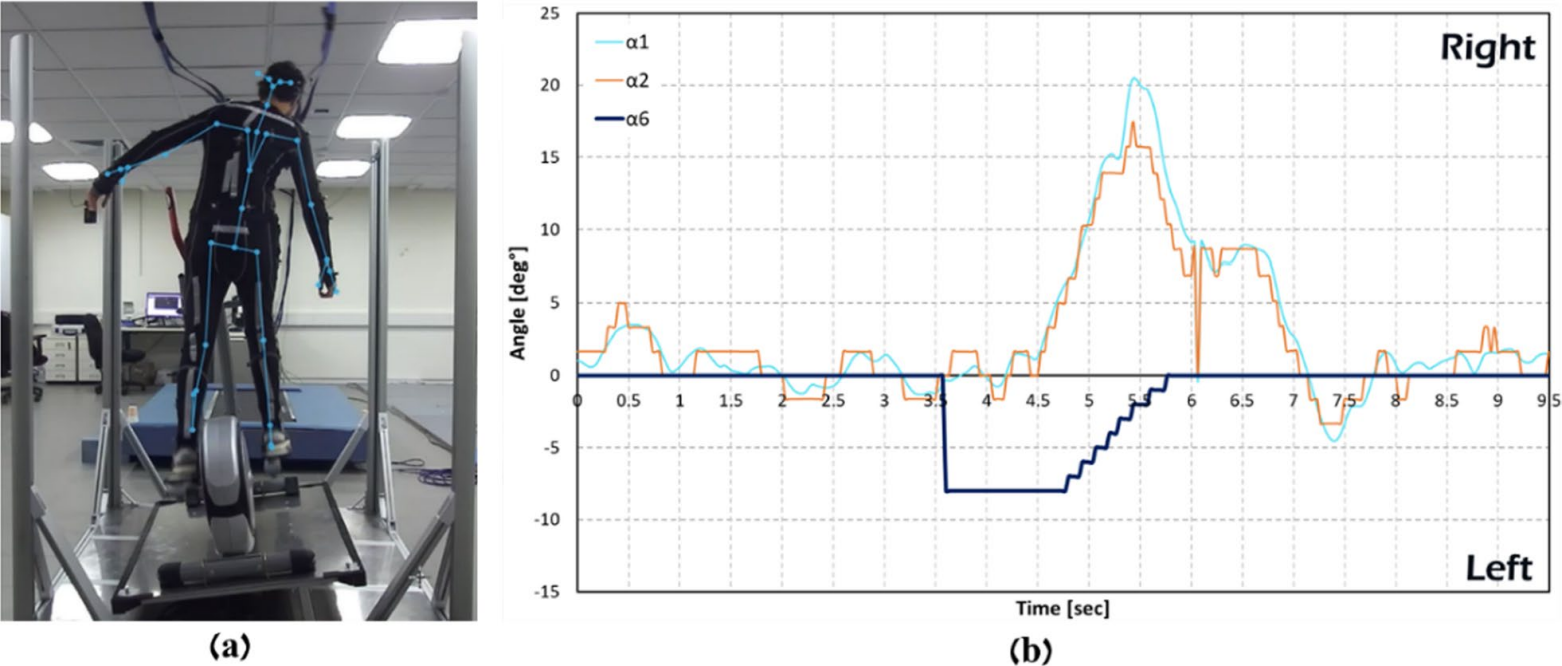
8° left tilt perturbation. **a** Camera image during the experiment, **b** a graph created from the saved CSV file. The system roll angle increases by 8° (dark line, *α6*), followed by a large balance recovery reaction as seen by about 17° right trunk angle (orange line, *α*2) and about 20° right shoulder angle (light blue line, *α*1) is, as clearly seen in **b**. Immediately after a good balance recovery response was detected by the software, the EPES system returns to its initial horizontal condition (dark line, *α*6)

In Fig. 8, the participant pedaled without holding the elliptical system handlebars, and the participant was exposed to 8° left announced perturbations during pedaling (Fig. 8A). Figure 8B shows that the EPES software was able to accurately identify shoulder and trunk balance reactive responses and return the system to its initial horizontal plane (Fig. 8B).

**Fig. 9:**
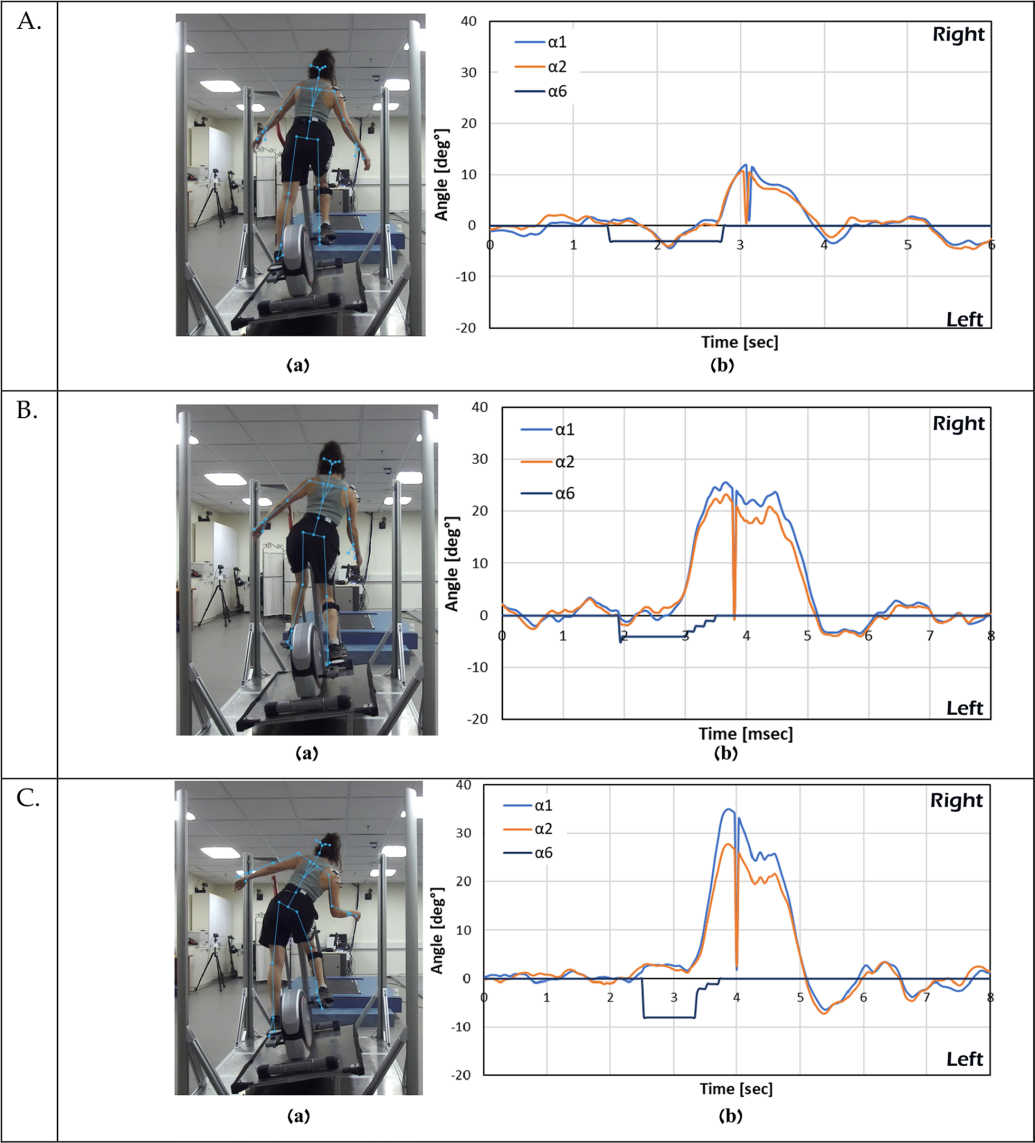
Example of an experiment: **a** camera image during the experiment, **b** a graph created from the saved CSV file. The system’s angle (*α*6) increases by 3° left tilt perturbation (**A**); 5° left tilt perturbation (**B**); and 8° left tilt perturbation (**C**). All perturbations are followed by a balance recovery reaction (angle *α*1 and *α*2) that was detected by the software, the EPES system returns to its initial horizontal condition as clearly seen (*α*6). There is a clear gradation in the participant balance recovery response based on the size of the disturbance

In Fig. 9A–C(a, b), the participant pedaled without holding the elliptical system handlebars and exposed to 3°, 5° and 8° left announced perturbations. Figure 9A–C shows that immediately post perturbation balance reactive responses were triggered to the opposite direction on perturbation, and that there is a clear gradation in the participant response (about 10°, 20°, and 30°), based on the size of the disturbance (3°, 5°, and 8° respectively).

Observing and analyzing a trainee’s balance reactive performance can be useful for making a clinical decision regarding the progress of rehabilitation, and for indicating skill acquisition and motor learning progress of the balance upper bodies reactive responses. Additional software enables the trainer to observe the kinematic data of a specific trainee in a specific training session. It presents kinematic graphs and a moveable timeline that allows the trainer to observe the upper bodies angles and the camera’s body stick figure at every timestamp, compared to the horizontal angle position of the EPES at that timestamp.

## Discussion

We found that the EPES system can evoke upper-body reactive responses i.e., trunk and arm balance responses. We also demonstrated that using a camera (ZED 2 from STEREOLABS), and development of the software that are able to detect upper-body balance proactive and reactive responses which controls the motors (i.e., return the EPES to its horizontal plane) and may enhance the motor learning process of reactive responses.

### Perturbation progress and clinical applications

Based on the EPES system’s features and our pilot results, and with respect to principals of motor learning, strength endurance, and especially balance control, we suggest a gradual increase in the difficulty of an EPES training program:

1. **No external support**—it was previously found that holding onto the handlebars can significantly reduce the postural reactive responses [54]. The main goal of any balance perturbation training is to evoke these balance reactive responses, thus, the trainee’s instructed to practice without holding the handlebars, i.e., hands-free training during elliptical walking. Since, the trainee’s feet were not strapped into the elliptical pedals, they are instructed to try to avoid stepping as much as they can. In case the trainee’s feel that he/she is unable to recover balance in fix of support strategy, they were instructed to hold/grasp the ellipticals handlebars.
2. **Overload and progressiveness**—the EPES programed so it can gradually increase the perturbation magnitudes (i.e., tilt, velocity, and accelerations of the moving platform).
3. **Random or block training**—the training program can be set to a block training (i.e., fixed time interval, specific direction, and specific magnitude) whereas the trainee is aware of the direction of the perturbations and the timing with a signal 5 s ahead of the perturbation. In addition, a random training (in onset, magnitude, and direction) can be programed. Varied practice in a random order was found to better improve motor learning [48, 49, 55, 56].
4. **Augmented feedback, implicit training**—once a balance perturbation is given, if appropriate balance reactive response is detected, the EPES returns to its horizontal, initial position. This intrinsic task feedback provides the trainee with an implicit cue for his/her successful balance response and provides the best possible motor learning implementation [48, 49].
5. **Overload**—the elliptical walking resistance can be also increasing along the training process, with the aim of improving endurance and lower limbs muscle power as well.
6. **Repetitions**—unannounced perturbations can be provided so that the participant will explore the best way to recover. The number of repetitions can be matched to the trainee’s abilities.

### Training session duration and training period

According to systematic review most perturbation training paradigms in standing or walking, were feasible for older adults includes and do not require long training periods for significant improvement of reactive balance responses and reduction in fall incidence [12]. Since the EPES system focuses on improving upper-body balance reactive responses by evoking upper-body balance responses. We suggest that each session last for 20 min, that includes 2-min self-paced warm-up pedaling (which is also the time frame for calibration) and 18 min of perturbation training for 12–20 training sessions.

### Target population

Generally, the target population consists of older people, in this case older adults with lower limbs osteoarthritis will be specifically targeted.

### Strengths

This is a novel intervention method of a technology that provides self-induced and unannounced perturbations during stationary elliptical walking, which is designed to improve balance function among older people. It touches on an important point in the field of fall prevention, as well as rehabilitation and motor learning principles—the ability to transfer balance recovery reactions that are acquired in a sitting position into a target context of balance control performances in standing and walking. All these components provide the best possible motor learning implementation of reactive balance response during walking and allow these exercises to be customized according to each trainee’s ability.

### Weaknesses

The EPES aims to improve balance control and balance reactive components by tilting perturbations. However, these perturbations during elliptical walking may not be similar enough to balance loss situations that cause real-life falls among older people; thus, they may not be specific. Because of the lack of specificity in this model, we may find no effects of the intervention on fall reduction. Our pilot includes quantitative measures of reactive balance during elliptical walking on the EPES but not in over ground standing and walking. However, our pilot results suggest that it is possible to evoke specific balance reactive responses in response to unexpected perturbation during EPES training thus it may improve balance reactive skills. It is still unknown if EPES training could prevent falls in older persons.

## Conclusions

This paper describes the EPES that provides programmed controlled small-to-high unannounced lateral balance perturbations during stationary elliptical walking. We showed that the software program designed specifically for the EPES was able to identify and analyze trainees’ balance reactive performance using our Camera and software. We also showed that, in a relatively short period of training time the trainee was able to perform correct/effective balance reactive responses, indicating that he was able to learn upper-body reactive balance responses. Furthermore, a future randomized controlled study should investigate whether older adults with osteoarthritis of the lower limb joints can improve their balance recovery responses, function in standing and walking, and reduce their rate of falls after EPES training.

## Data Availability

Not applicable.

## Abbreviations

EPES: **E**lliptical **Pe**rturbation **S**ystem
CoM: Center of mass
